# Modeling Supply and Demand Dynamics of Vaccines against Epidemic-Prone Pathogens: Case Study of Ebola Virus Disease

**DOI:** 10.3390/vaccines12010024

**Published:** 2023-12-25

**Authors:** Donovan Guttieres, Charlot Diepvens, Catherine Decouttere, Nico Vandaele

**Affiliations:** Access-to-Medicines Research Centre, Faculty of Economics & Business, KU Leuven, 3000 Leuven, Belgium; charlot.diepvens@kuleuven.be (C.D.); catherine.decouttere@kuleuven.be (C.D.); nico.vandaele@kuleuven.be (N.V.)

**Keywords:** vaccines, immunization, infectious diseases, epidemics, sustainability, Ebola Virus Disease, filoviruses, system dynamics

## Abstract

Health emergencies caused by epidemic-prone pathogens (EPPs) have increased exponentially in recent decades. Although vaccines have proven beneficial, they are unavailable for many pathogens. Furthermore, achieving timely and equitable access to vaccines against EPPs is not trivial. It requires decision-makers to capture numerous interrelated factors across temporal and spatial scales, with significant uncertainties, variability, delays, and feedback loops that give rise to dynamic and unexpected behavior. Therefore, despite progress in filling R&D gaps, the path to licensure and the long-term viability of vaccines against EPPs continues to be unclear. This paper presents a quantitative system dynamics modeling framework to evaluate the long-term sustainability of vaccine supply under different vaccination strategies. Data from both literature and 50 expert interviews are used to model the supply and demand of a prototypical Ebolavirus Zaire (EBOV) vaccine. Specifically, the case study evaluates dynamics associated with proactive vaccination ahead of an outbreak of similar magnitude as the 2018–2020 epidemic in North Kivu, Democratic Republic of the Congo. The scenarios presented demonstrate how uncertainties (e.g., duration of vaccine-induced protection) and design criteria (e.g., priority geographies and groups, target coverage, frequency of boosters) lead to important tradeoffs across policy aims, public health outcomes, and feasibility (e.g., technical, operational, financial). With sufficient context and data, the framework provides a foundation to apply the model to a broad range of additional geographies and priority pathogens. Furthermore, the ability to identify leverage points for long-term preparedness offers directions for further research.

## 1. Introduction

Recent emerging and re-emerging infectious disease outbreaks, including the COVID-19 pandemic (COVID-19), have exposed our vulnerability to unforeseen public health emergencies and present a growing threat to society [1]. They have the potential to rapidly overwhelm health systems, disrupt routine services, provoke fear, and unleash catastrophic socioeconomic costs. Since 1960, epidemics caused by wildlife zoonoses have increased exponentially [2], especially in resource-constrained geographies such as Sub-Saharan Africa [3]. Infectious disease outbreaks often interact with and exacerbate existing socio-economic inequities, with elevated exposure and transmission in the most susceptible populations [4]. The rise in epidemics can partly be explained by changing demographic patterns, with urbanization accelerating the spread of disease and increasingly encroaching on zoonotic reservoirs [5]. Furthermore, increased mobility from global travel, migration, and international wildlife trade displaces pathogens across geographic and ecological boundaries [6,7]. Finally, anthropogenic climate change has been shown to aggravate more than 50% of infectious diseases, for example by enhancing pathogen virulence [8]. Despite renewed pandemic preparedness efforts, insufficient integration across health systems and a high level of uncertainty around future risks jeopardize sustained, scalable, and equitable responses [9]. 

The lack of integration across response efforts was especially evident during COVID-19, with numerous concurrent challenges contributing to large coverage gaps even as supply became abundant. These include hoarding of raw material supplies, border restrictions, the emergence of novel viral variants, and large inequities in access aggravating vaccine hesitancy [10]. Building an end-to-end ecosystem that ensures timely and equitable access to medical countermeasures is a critical step toward meeting local public health needs [11]. Historically, vaccines have played a significant role in promoting health, as well as generating social and economic value [12,13]. The COVID-19 vaccines prevented over 14 million deaths in the first year of administration, although more could have been averted with more equitable allocation [14]. Furthermore, although non-pharmaceutical interventions (NPIs) help reduce disease spread, they are not always sufficient and lead to unintended consequences (e.g., economic recession, decline in mental health) depending on their stringency and behavioral response [15,16]. 

Despite large benefits, vaccines are unavailable for many pathogens that pose a severe threat to society (e.g., Lassa, Marburg, Zika) [17]. To address this gap, since 2016, the World Health Organization (WHO) has published and updated a list of priority diseases requiring R&D attention, based on epidemic-prone pathogens (EPPs) for which there are no or insufficient countermeasures [18]. This includes ‘Disease X’, referring to unknown pathogens that could cause public health emergencies following zoonotic spillover into human populations. R&D roadmaps have also been defined to promote coordination around these priority diseases and incentivize accelerated vaccine development. Beyond the typical time- and resource-intensive product development process, market-shaping mechanisms are important to overcome the limited commercial viability that comes with sporadic and limited demand [19]. Ensuring continued investments in the development of vaccines against EPPs requires novel strategies that promote sustainability and coordination [20,21]. Initiatives such as the Coalition for Epidemic Preparedness Innovations (CEPI) were established to de-risk vaccine R&D, for example by supporting the development of technology platforms, clinical trials, and regulatory pathways to reduce response time to future outbreaks [22]. 

Although much progress has been made to fill the R&D gap, the path to licensure and the long-term viability of vaccines against EPPs continues to be unclear. The sporadic nature of outbreaks makes it logistically challenging and ethically questionable to conduct large clinical trials [23,24], leading to uncertainty around key clinical parameters such as vaccine effectiveness and duration of vaccine-induced immunity. This uncertainty makes it difficult to provide clear guidance on the most appropriate and effective use of late-stage or licensed vaccines during both outbreak and inter-epidemic settings. As a result, defining an investment case becomes challenging for various stakeholders, be it national governments or international organizations such as Gavi (the Vaccine Alliance), aiming to support programmatic implementation. The typical reactive approach to public health emergencies further complicates preparedness efforts. Uncertainty in the timing, location, and spread of outbreaks [25], as well as the evolution of pathogens, makes it difficult to plan and leaves at-risk populations vulnerable to disruptions across the supply chain [26]. Although emergency stockpiles have been established to ensure rapid deployment of vaccines when outbreaks emerge, demand uncertainty makes it difficult to initially size and continuously maintain stock levels while minimizing both shortages and wastage [27,28]. Finally, the political, economic, and sociocultural context in which outbreaks emerge, including competing health priorities, are complex and need to be adequately considered for sustained preparedness. 

Addressing these challenges requires a global understanding of the complex network of interdependent stakeholders, processes, and decision points involved. Stakeholders often have varying goals and resources, with silos leading to fragmentation and insufficient coordination [1]. In this context, achieving timely and equitable access to vaccines against EPPs is not trivial. It requires decision-makers to capture numerous interrelated factors across temporal and spatial scales, with significant uncertainty, variability, delays, and feedback loops that give rise to dynamic and unexpected system behavior. Therefore, this paper presents a quantitative system dynamics modeling framework that integrates numerous stakeholder perspectives and factors on both the supply and demand side. It demonstrates how modeling can help learn about system behavior and assess the long-term sustainability of vaccine supply under different demand scenarios, based on user-defined vaccination strategies. Specifically, sustainability is evaluated from multiple dimensions: (1) country-level vaccine availability, (2) protection of at-risk populations, and (3) investment requirements, incentives, and priorities. 

The remainder of the paper is structured in the following manner: Section 2 presents a case study of Ebolavirus Zaire (EBOV), Section 3 provides an overview of the methodology for building and validating the model, Section 4 outlines each subsystem considered, Section 5 presents baseline model results and sensitivity analysis for different leverage points in the system, and Section 6 outlines learnings, limitations, and future research directions.

## 2. Case Study: EBOV

Ebola virus disease (EVD), a highly fatal and severe illness that causes hemorrhagic fever following infection from viruses in the *Filoviridae* family, was first reported in 1976 in the Democratic Republic of the Congo (DRC) [29]. Since then, there have been recurring EVD outbreaks across Central and West Africa, known to be zoonotic reservoirs for these viruses. EBOV, a single-stranded RNA virus with a genome that encodes for 7 proteins [30], is by far the most common virus causing EVD. The surface glycoprotein (GP) plays an important role in immune evasion and disease progression, making it a key antigenic target for the development of Ebola vaccines [31]. Although the natural reservoir of EBOV is believed to be fruit bats, various animals such as non-human primates serve as intermediate hosts that can shed the virus and infect humans that encounter them, for example through hunting [31].

The worst EVD outbreak, also known as the West Africa Ebola epidemic (2014–2016), led to more than 28,000 cases, 11,000 deaths, and an economic cost of around USD 53 billion in the region [32]. Recognizing the threat posed by EBOV outbreaks, the international community accelerated the development of diagnostics, vaccines, and treatments. Furthermore, the International Coordination Group (ICG) on vaccine provision established a 500,000-dose emergency stockpile in 2021. With USD 178 million in funding by GAVI, the stockpile facilitates rapid deployment of vaccines to affected countries when future outbreaks emerge [33]. 

Currently, two vaccines have received WHO pre-qualification against EBOV, while many more are in development for other viruses in the *Filoviridae* family such as the Sudan species of Ebola (SUDV) and Marburg (MARV) [34]. Ervebo, initially developed by the Public Health Agency of Canada before being licensed to Merck, is a one-dose replication-competent viral vector vaccine that consists of a live attenuated recombinant vesicular stomatitis virus (rVSV). Zabdeno/Mvabea, developed by Janssen, is a prime-boost heterologous replication-incompetent viral vector vaccine regimen. Although these vaccines demonstrate a protective effect by generating antibody titers, there continues to be limited real-world evidence on effectiveness due to the sporadic nature of outbreaks and ambiguous correlates of immune protection [35]. Furthermore, each vaccine has unique properties that lead to diverging use cases and supply requirements.

The post-license use of these vaccines is guided by the Strategic Advisory Group of Experts (SAGE) working group on Ebola vaccines and vaccination. As of the last SAGE update in March 2021, both vaccines are recommended for reactive use in outbreak settings following a confirmed case of EBOV [36]. Specifically, Ervebo can be deployed under a ring vaccination protocol, while a “geographically targeted vaccination strategy may be considered when it is impossible to identify the individuals who make up ring vaccination cohorts because of serious security, social or epidemiological issues” [37]. Zabdeno/Mvabea is recommended for those at “some, but lower, risk of EVD in the context of an outbreak, such as health workers and frontline workers, in neighboring areas and countries where the outbreak may spread” [37]. Due to supply constraints at the time, these recommendations aim to curb outbreaks in a dose-sparing manner. As the ICG stockpile reached its target level at the end of 2022, vaccines approaching expiry can now be accessed for proactive vaccination of at-risk groups [38]. 

Although vaccinating 16,000 people in Guinea and 300,000 in eastern DRC during the two largest EVD outbreaks in history helped control transmission, these epidemics continued for several years [39]. As seen in previous outbreaks, reactive ring vaccination relies heavily on effective contract tracing to cut transmission chains [40]. This is particularly challenging when mobility or deliberate avoidance is present, leading to unmonitored transmission, on top of already-existing logistic factors (e.g., reaching remote or conflict areas) [41,42]. These challenges, as well as the recurrence of EBOV outbreaks, point to the need for proactive vaccine strategies in high-risk geographies and groups, to mitigate the onset and spread of disease [39,43]. 

**Figure 1:**
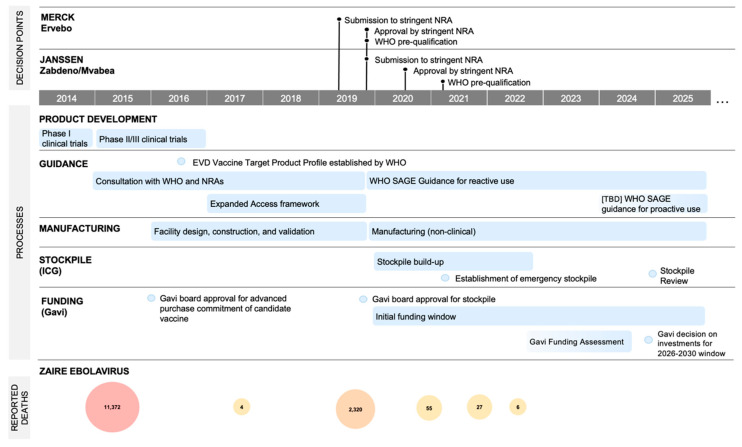
Key decision points and processes related to WHO pre-qualified vaccines against EVD since the 2014–2016 West Africa Ebola epidemic, including reported deaths [45] from historic EBOV outbreaks since 2014.

A complex network of processes and decision points (see Figure 1) could cause significant delays in timely access to vaccines. This inaction jeopardizes the ability to effectively prepare for future outbreaks. In 2022, EVD was the highest-ranked emerging infectious disease threat in a report to inform the African Union’s strategic planning around emergency preparedness and response [44]. The growing experience around previous outbreaks and the use of vaccines provides a foundation to explore and compare alternative vaccination strategies. Nevertheless, reaping the full benefits of preparedness efforts relies on ensuring the sustainability of EBOV vaccines as public health needs evolve. 

## 3. Methodology

### 3.1. Study Aim

The purpose of the case study is to demonstrate the application of a modeling framework presented in this paper. More specifically, the case study is used to develop a dynamic hypothesis for the underlying factors driving the sustainability of EBOV vaccine supply. To achieve this, a model integrating both supply and demand dynamics was developed and validated using historical data. This provides a basis for simulating future scenarios and testing alternative system structures. Although two WHO pre-qualified vaccines exist, their unique and different properties make comparability difficult, while the effect of mixing and matching vaccines within the same population is unclear. Therefore, simulation results presented focus on the overall effect of a prototypical EBOV vaccine given different vaccination strategies, rather than individual assessment of currently licensed products. However, experience from both Ervebo and Zabdeno/Mvabea does provide a broader range of realistic parameter values to assess the impact of product design on other subsystems. Similarly, the results focus on exposing systems-level tradeoffs that emerge for different vaccination strategies rather than defining optimal interventions. 

### 3.2. Study Setting

Stella Architect (version 3.3), a software from Isee Systems, was used to build the model and run simulations. It supports a broad range of modeling capabilities, including continuous-time and discrete event components. The software provides a programming language to write mathematical equations graphically represented by four types of interlinked building blocks (stocks, flows, auxiliary variables, and parameters/constants) needed for system dynamics modeling [46]. At each time step, numerical integration based on finite difference equations is used to solve sets of differential equations to approximate model values [47]. To promote interoperability across simulation environments, models built in Stella Architect can be exported and converted into scripts to be manipulated in other programming languages such as R and Python. 

The primary scope of the model is the transition of EBOV vaccines from late-stage development to post-licensure, including establishing supply mechanisms aimed at achieving timely and equitable access under different vaccination strategies. The simulation period is 12 years, starting in March 2019 when the first EBOV vaccine (Ervebo) was submitted to a regulatory authority (European Medicines Agency) for approval, with a time step of 0.5 days. Given the inherent challenge of predicting the timing and scale of future outbreaks, historical data from the DRC, specifically the 2018–2020 epidemic in North Kivu, was considered. Given sufficient contextual knowledge and data, the modeling framework provides flexibility for users to explore various questions related to the supply and availability of EBOV vaccines, as well as consider a broader range of vaccination strategies, time horizons, geographies, and pathogens.

### 3.3. Modeling Approach

To facilitate strategic decision-making within complex, adaptive systems, system dynamics (SD) has shown to be a useful tool. SD is a computer-based mathematical modeling approach rooted in feedback theory and well-suited to simulate problems with time-varying behavior [48]. It has been applied to a wide range of healthcare problems, including modeling control strategies and disease dynamics for COVID-19 [49,50,51], influenza [52,53], polio [54], and Ebola [55,56,57], among other infectious disease outbreaks. Several studies have used SD to consider the impact of polio outbreaks following eradication on stockpile needs [58,59], while others have used optimization-based methods to inform supply planning of COVID-19 [60,61] and monkeypox [62] vaccines under demand uncertainty. However, models focused on production and distribution networks may not adequately capture the underlying drivers that give rise to dynamic behavior. Additionally, models integrating both the supply and demand of vaccines against EPPs are limited; none were found for EBOV. As infectious disease outbreaks become more frequent, designing systems to ensure the long-term sustainability of preparedness efforts is paramount.

Qualitative causal loop diagrams (CLDs) provide an overview of mechanisms driving behavior by mapping interactions between key variables found in data. CLDs serve as a starting point for quantitative modeling. In this work, SD is used to quantify the aggregate relationship between model variables and outputs of interest to generate insights on plausible scenarios rather than make specific predictions [63]. By capturing interdependencies among variables, SD sheds light on the complexities, delays, and unintended consequences inherent in systems. Of particular relevance, SD provides an endogenous understanding of observed behavior [64], in part by overcoming limited mental models of problems that arise from siloed disciplines or subjective experiences [65]. This is done by incorporating broad stakeholder perspectives throughout the process, from problem identification to scenario definition. The methodology builds on previous work centered on iterative systems and stakeholder analysis to develop models that can aid in decision support [66,67].

### 3.4. Data Collection and Analysis

**Figure 2:**
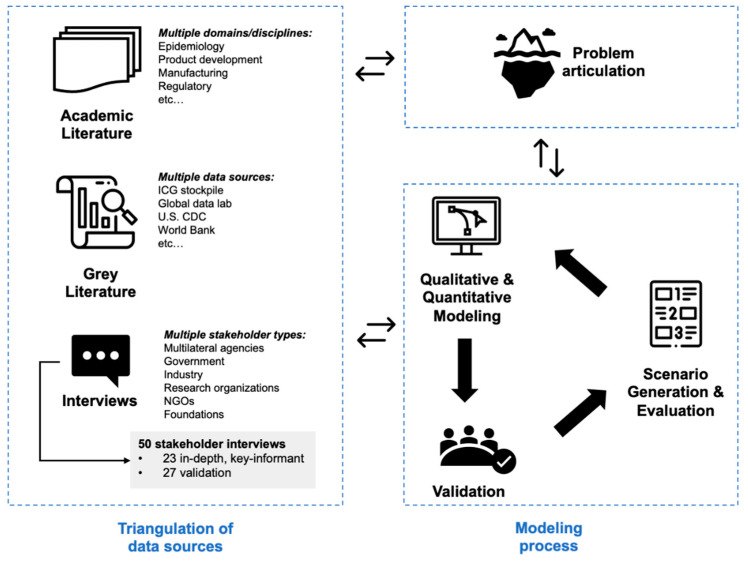
Iterative methodology focused on problem articulation, triangulation of data sources, and modeling.

To formulate a model that accurately reflects supply and demand dynamics of EBOV vaccines, as well as assess relevant leverage points, this research triangulated academic literature, grey literature, and interviews (see Figure 2). An iterative data collection process was followed, starting with academic and grey literature. This was complemented with one-hour, semi-structured interviews conducted in French or English, depending on the participant’s preferred language. As part of the research protocol, each interviewee signed an informed consent form expressing their preference for pseudonymizing information used from interviews. Recordings and transcripts of interviews were securely stored and deleted post-transcription to ensure data privacy and confidentiality. When information was missing from the consent form, the authors assumed the most stringent level of anonymization. In total, 50 expert interviews were conducted with a broad range of stakeholders. Of these, 23 were key-informant and in-depth interviews using a pre-defined set of questions (see interview guide in Supplementary A), focusing on topics most closely aligned with each interviewee’s expertise. An additional 27 validation interviews were conducted to fill knowledge gaps and solicit feedback on model structures, iteratively improving the model (see interview guide in Supplementary B). 

Both literature and interviewees were identified using a snowballing strategy. Initially, stakeholders were chosen based on identified gaps in the existing literature. Subsequently, recognizing the diverse and partial nature of the informant’s views on the problem, researchers aimed to balance perspectives by categorizing stakeholders according to their organization type, as outlined in Supplementary C. Interviewees were required to have at least three years of expertise in immunization and/or infectious disease outbreaks, although most had 10+ years of relevant work experience. The interview process continued until it reached a point of saturation, to the authors’ knowledge, indicating no further novel insights relevant to the model scope were obtained. Data from literature and interviews was used to inform the logic behind model structures, identify realistic values for uncertain parameters, and define scenarios for vaccination strategies. Other techniques used to estimate parameters include historical data, heuristics, and stochastic distributions. 

### 3.5. Model Validation

Validation tests were first conducted for each subsystem individually and repeated for the fully integrated model. The unit consistency of the model was first checked. Various model structure and behavior tests were then conducted [68]. In some cases, when published data was limited, the validity of model parameters, structures, and behavior was checked with stakeholders during interviews, based on their area of expertise. For model equations, as well as table and graphical functions, different input values were tested to ensure output followed the expected behavior. Extreme condition tests were used to assess model behavior which may not otherwise be seen in the baseline. Behavior reproduction compared model outcomes to key reference modes defined by stakeholders or based on historical data. Although these tests help build confidence in model insights, it is important to reiterate the aims of the results presented: (1) illustrating the value generated from an SD modeling framework, and (2) building a greater understanding of system behavior for strategic decision-support. As such, the model is not meant for specific predictions or to define optimal operations. Users should be especially aware of the unique contextual factors driving behavior and adapt model inputs to better capture knowledge accumulated over time. 

### 3.6. System Definition 

The supply of vaccines to protect against EBOV, whether reactively following an outbreak or proactively during inter-epidemic periods, presents unique challenges compared to routine immunization. Since little knowledge is available on the nature of future outbreaks before they happen, supply systems need to adapt as information becomes available and risks evolve. Despite this uncertainty, a core function of the immunization system is ensuring timely access to high-quality, effective vaccines to protect at-risk populations and halt disease transmission. Given the many interdependencies across the system, a better understanding of major subsystems influencing the timely supply of vaccines against EPPs is needed to adequately meet local public health needs. This involves capturing each subsystem’s stakeholders, functions, interdependencies, and structural limitations. Although there is extensive work done by domain experts on the dynamics within individual subsystems, addressing systemic risks and uncertainties requires system-level analysis [69]. 

**Figure 3:**
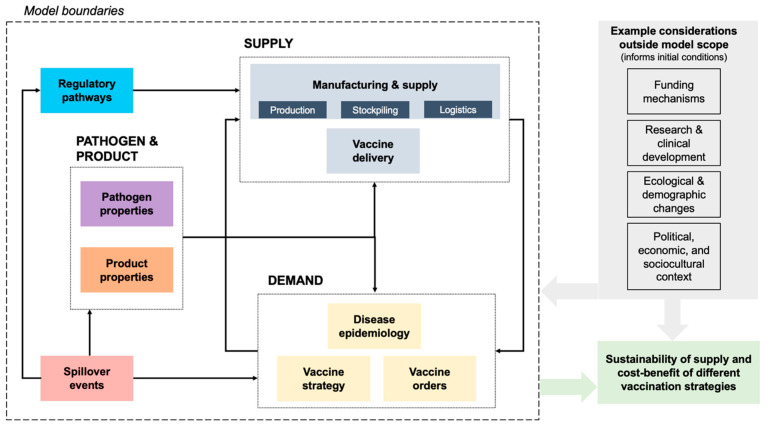
Overview of subsystems considered within model boundaries leading to supply–demand interactions, as well as key considerations beyond model scope that inform baseline conditions.

System boundaries are defined by the research aim, as they need to capture factors driving the behavior being investigated. The inclusion of relevant factors and their dependencies is based on extensive stakeholder interviews and published literature. An overview of subsystems considered in the modeling framework is presented in Figure 3, with a more detailed explanation found in Section 4. Similarly, the extent to which phenomena within each individual subsystem are endogenously modeled depends on (1) feasibility (availability of data or knowledge) and (2) whether more details would lead to different insights with respect to the research aim. 

### 3.7. Model Application

Depending on the specific research question, the modeling framework can be used to evaluate a broad range of interventions at different leverage points in the system. For complex systems, a unique value of SD models is the ability to intervene at different levels. The choice of leverage point has implications on the resources required and how effectively it can change system behavior. At one extreme, low-leverage points (e.g., variations in parameter values, length of delays) can be significant in the short term but are rarely sustained to change long-term behavior, whereas high-leverage points (e.g., incentives, system goals, paradigms) lead to fundamental changes in system structure that give rise to novel outcomes [70]. 

**Figure 4:**
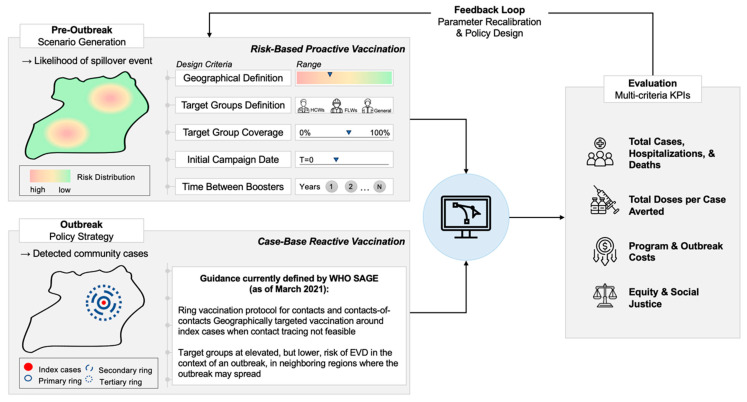
Given an established policy for case-based reactive vaccination against EBOV, workflow for multi-criteria evaluation of user-defined scenarios for proactive vaccination against epidemic-prone pathogens.

The core application presented in this paper is assessing system-level tradeoffs associated with shifting from reactive to proactive vaccination strategies for vaccines against EPPs. The use of vaccines in non-outbreak settings requires the assessment of multiple design criteria, as seen in Figure 4. This can quickly lead to a complex, multi-dimensional scenario space that will influence a broad range of key performance indicators (KPIs) that directly impact the sustainability and long-term value of the vaccination strategy considered. The model allows simultaneous assessment of multiple types of KPIs (e.g., physical, financial, value-based), reflecting different stakeholder perspectives which often need to be reconciled to promote sustainability in access [66]. Although it can be used to evaluate a much broader set of scenarios, a few illustrative examples are presented in Section 5.

## 4. Model Subsystems

Although not exhaustive, this section defines the scope for each subsystem included in the modeling framework. It introduces relevant parameters directly influencing supply and demand dynamics of EBOV vaccines. When relevant, alternative subsystem-specific modeling techniques and possible extensions are presented. More information on parameters, assumptions, and baseline values used to generate model results are found in Supplementary D. For the most part, the model captures key technical processes and decision points that drive system behavior. Integrating these subsystems into a common framework expands the boundaries to better capture causal pathways between subsystems leading to dynamic behavior. Depending on the specific model setting, users can further define the specific political, economic, and sociocultural context.

### 4.1. Pathogen Properties

While numerous pathogens could lead to catastrophic health emergencies, viruses are the most likely to cause epidemics given their relatively high rate of replication and accumulation of mutations. Around 260 viruses from 25 families are known to infect humans, though more than 1.5 million additional viruses are believed to exist in various zoonotic reservoirs [71]. Despite the large number of individual viruses, those within the same viral family share common functional and structural properties. Several characteristics that drive epidemic risks include zoonotic shifts, modes of transmission, and viral adaptation to propagate within human populations [72].

Studies have shown a high degree of conservation (~97–98%) between EBOV strains, even for those with large spatial and temporal spread [73], though this is much lower (~60–65%) when comparing GP sequences across Ebola species [74]. This indicates that first-generation vaccines could provide protection against future EBOV outbreaks, though would not be broadly protective against other species such as SUDV. Sequencing of clinical samples from previous EBOV outbreaks shows mutations contributing to enhanced viral fitness, infectivity, and case-fatality rate (CFR) [75,76]. However, there continues to be limited understanding of the extent to which viral evolution, either through natural selection or genetic drift, influences the efficacy of EBOV vaccines as demonstrated for sequence-based therapeutics [77,78]. Although the risk of viral variants is accentuated during large, extended outbreaks, it does not necessarily reflect the evolutionary rate of EBOV and its potential long-term impact on vaccines.

### 4.2. Spillover Events

The sporadic nature of infectious disease outbreaks presents a continued public health challenge. Anticipating the location and timing of potential future spillover events is important for more targeted surveillance. Among viruses in the *Filoviridae* family, EBOV has been the most frequent and fatal, with 24 outbreaks since 1976 (seven of which have occurred since the 2018–2020 epidemic in the DRC) and over 30,000 deaths [45]. Although most outbreaks have been small, they are increasingly moving from hard-to-reach, rural, and conflict zones to more urban areas that could accelerate the spread of future epidemics. In this model, spillover events are defined exogenously. Users can determine the specific timing (day) and location (at the country or sub-national level) of future index cases to then model potential trajectories of the outbreak. This includes specifying the number of individuals initially exposed to EBOV, as well as the timing of imported cases to both neighboring countries and more distant regions, as experienced during the 2013–2016 West Africa Ebola epidemic.

In further research, model boundaries could be extended to endogenously account for underlying drivers of spillover risks. This would require a deeper understanding of the ecological context and reservoir hosts in which EBOV evolves. Analysis of historical index cases points to tropical forests as suitable habitats for EBOV and elevated risk from contact with bushmeat [79]. Additionally, various geographic and statistical models have been developed to identify risk factors and forecast spillover intensity under different spatial, temporal, and socioeconomic contexts [80,81,82,83,84]. These models point to elevated risks in countries with historic outbreaks, as well as the broader zoonotic niche of EBOV, and could serve as a good foundation for more extensive modeling within this subsystem.

### 4.3. Disease Epidemiology

Modeling the spread of infectious diseases following an outbreak is important for effective resource planning and strategic use of countermeasures, especially since decision-makers need to act rapidly despite limited knowledge [85]. Typically, disease transmission models have been developed by assigning disease states to populations and defining transitions based on various epidemiological parameters (e.g., incubation period). Depending on the type and extent to which data is available, various modeling techniques can be employed. Regardless of the approach, common challenges exist, such as capturing the inherent uncertainty of future outbreaks—both from limited knowledge of the system and observational error or bias [86]. Techniques for assessing model uncertainty include (1) generating multiple outputs of a deterministic model by varying input parameter values, and (2) building stochastic models that inherently incorporate variability in parameters [87]. Reporting model assumptions and limitations, or adapting model structures to reflect local realities, is key.

Numerous and diverse transmission models have been developed for EVD, allowing users to explore different techniques based on their specific research questions and preferences. These include individual, network-based transmission models using techniques such as pair approximation and branching processes to assess the impact of ring vaccination [40,88,89], as well as a mix of mathematical, compartmental, and agent-based models to evaluate community vaccination [90,91,92]. The stock-and-flow structure of SD is particularly well suited for the simulation of disease transmission using mean-field compartmental models. Generally, the population is divided into different states, such as: susceptible (S), exposed (E), infected (I), or recovered (R). Numerous variations of the SEIR model exist, with additional compartments, depending on the level of detail included and the specific behavior being modeled. At each time point, individuals can only occupy one stock and progress sequentially from one state to the next. The rate of transition between each state is governed by a set of partial differential equations that capture the time individuals are expected to stay in a given stock. Depending on the virus, severity of infection, and strength of the natural or induced immune response, an individual can revert to the susceptible state if they lose immunity over time.

For EVD, a common model formulation expands on the SEIR structure to differentiate transmission due to contact with (1) infected individuals in the community after onset of symptoms (I), (2) infected individuals that are then hospitalized (H), and (3) infected or hospitalized individuals that die from EVD but have not been buried or during traditional funerals [93]. Individuals in both the I and H stocks can either recover or die (and eventually get buried). Other models account for these three transmission modes and split the susceptible population (S) into healthcare workers (HCWs) and the rest of the general population (non-HCWs) given their different exposure to risk [91,92,94]. The model structure and parameter estimations presented in Potluri et al. (2022) serve as a basis for the epidemiologic subsystem. Using least squares estimation techniques, time-dependent transmission rates and other parameters are calibrated to WHO-reported cases and deaths from the 2018–2020 EVD epidemic in North Kivu to reflect real-world disease dynamics.

However, several conceptual and structural extensions were made: (1) stochastic simulations were done in an SD software rather than the direct method algorithm described in Gillespie (1976) [95]; (2) in addition to accounting for potential infection of vaccinated (prior to the onset of immunity) and protected (after the onset of immunity) individuals, the model also considers loss of immunity over time; (3) account for the relative timing between vaccination campaigns and onset of EBOV outbreaks, since immunity from vaccination may fade before the next outbreak starts; (4) beyond the target vaccination coverage rate for HCWs and non-HCWs population, various parameters that can delay vaccination campaigns (e.g., case detection and reporting) and limit uptake (e.g., vaccine hesitancy) are considered; and (5) demand signals defined by vaccination strategies are linked to manufacturing and supply operations.

### 4.4. Vaccine Strategy and Country Orders

Vaccination confers protective immunity against pathogens before encountering them. In the context of infectious disease outbreaks, vaccination campaigns can be conducted reactively in active (or neighboring) outbreak regions or proactively during inter-epidemic periods. Vaccination strategies should be designed to achieve specific aims. Broadly defined, public health programs have aimed to achieve one of the following: eradication (global absence of human cases and zoonotic reservoirs), elimination (location-specific cessation of transmission), and control (restricting pathogen circulation below a pre-defined level) [96]. Given the uncertainty in the natural host, EVD control seems most realistic. However, a challenge comes from the heterogeneity of spillover risks and response capabilities across geographies. Therefore, a global vaccination strategy needs to be designed in a way that can adapt to asynchronous public health needs across different countries and contexts, while staying operationally feasible and cost-effective over time. Given the already-constrained capacities in many EBOV-endemic countries and the ability for outbreaks to rapidly disrupt routine services [97,98], vaccination strategies should aim to reduce cases and hospitalizations to avoid saturation and subsequent collapse of the healthcare system of affected areas.

Decision-makers are faced with many options when designing vaccination programs to meet strategic aims. Based on EVD risks, they need to determine which specific subgroups to vaccinate and specify the target coverage for each. In Liberia, 0.11% of the general population compared to 8.07% of healthcare workers died during the 2014–2016 West Africa Epidemic [99]. The concentration of incidence and deaths among HCWs has detrimental effects on maternal, infant, and under-5 mortality in affected countries [100]. Moreover, elevated seroactivity in asymptomatic HCWs in the DRC also points to elevated exposure to EBOV relative to the general population [101]. With the continued shortage of HCWs in many EBOV endemic countries, frontline staff, and high-risk groups (e.g., burial teams, religious leaders, drivers) could be prioritized for vaccination in parallel to standard practices. Another sub-population to consider is survivors and their contacts, as there is evidence of viral persistence for up to two years in immune-privileged sites leading to clinical relapse and sexual transmission [102,103,104]. Although the risks are not well defined, viral persistence-derived transmission of EBOV points to the importance of increased surveillance and tracing in regions with prior known or undetected transmission [105]. Another consideration is around the geographic focus of vaccination. Spatial-temporal analysis can help overcome reliance on contact tracing seen in ring vaccination and classify regions based on future spillover risks, as well as spread to neighboring areas [88,106]. The timing and frequency of vaccination relative to future EBOV outbreaks is also important, especially when immunity wanes over time. Since the model simulates behavior over long periods, it captures population dynamics such as migration in a region to reflect immunity levels more accurately in the target groups.

The design considerations described above have a direct impact on the number of vaccines ordered by countries over time, serving as a demand signal to plan corresponding supply-side operations. However, even when a vaccination strategy has been defined, numerous factors can delay vaccine orders, for example: SAGE recommendations, regulatory review by countries that have yet to license vaccines, mobilizing resources, and setting up campaigns. Vaccination strategies define a population’s immune protection over time and have the potential to promote equity (e.g., prioritizing the highest-risk groups). The complex sociocultural and political context in which EBOV outbreaks emerge points to the importance of community engagement, effective communication, and building trust to successfully implement control measures [107,108,109].

### 4.5. Product Properties

Vaccine product properties are strong drivers of potential benefits relative to the risk of EVD exposure. To encourage product development and improve awareness around criteria for assessing candidate products, the WHO published in 2016 a vaccine target product profile defining preferred characteristics for both reactive and proactive use of EBOV vaccines [110]. Factors such as the number of doses, presentation, packaging, and thermostability have a significant impact on capacity requirements for manufacturing, supply, and last-mile delivery. For the most part, properties defined on the product label will not change much over time. However, in some cases, post-approval monitoring studies can lead to expanded use to different age groups or special populations (e.g., immunocompromised, pregnant women). Factors such as eligibility criteria are important as they can influence vaccination strategies and the design of vaccination campaigns. Although product improvements such as shelf-life extension will likely take significant time, the model can show the relative impact of alternative product properties on model behavior.

More specifically, both Ervebo and Zabdeno/Mvabea are packaged and stored at ultra-cold temperatures (−80 to −60 °C), giving them the longest possible shelf-life. However, since EBOV outbreaks are endemic in countries with limited cold chain infrastructure, introducing novel vaccines in a national IMS can add a burden to local capacities and compete with routine immunization [111]. Therefore, careful planning is needed to avoid temperature excursions and expiry after vaccines are thawed. The degree to which vaccines help control future outbreaks will also greatly depend on the time for vaccinated individuals to reach peak protection, vaccine effectiveness, duration of vaccine-induced immunity, and recommended time between boosters. Finally, adverse events also complicate vaccination efforts as they are difficult to distinguish from symptomatic EVD patients and cause fear, serving as the most common source of concern limiting uptake among HCWs in the DRC [112].

### 4.6. Regulatory Pathways

Depending on the epidemiologic context, various regulatory pathways exist to facilitate access to vaccines. In an ideal setting, clinical safety (Phase I and II) and efficacy (Phase III) studies are conducted to generate evidence to support full licensure of a vaccine. When infectious disease outbreaks are large and persistent, it may be feasible to set up Phase III trials, as was the case during the 2014–2016 Ebola epidemic in West Africa. More specifically, a cluster-randomized ring vaccination trial was conducted in Guinea for Ervebo, demonstrating strong efficacy as there were no cases among vaccinated individuals from day 10 after vaccination [113]. In most cases, however, the unpredictable and sporadic nature of viruses in the *Filoviridae* family makes it very challenging to set up large clinical trials and generate strong real-world evidence on the strength and duration of protection. Therefore, clinical trial designs need to consider the evolution of outbreaks, such as waning transmission over time and pre-existing immunity in the population, as well as delays in obtaining all relevant ethical and procedural approvals [114]. Since clinical trials may not always be ethical or feasible, a licensing pathway was developed to bridge animal efficacy data with human safety and immunogenicity data, assuming the pathophysiology of the animal model used closely resembles that of human disease [115]. For Zabdeno/Mvabea, immunobridging was used to correlate EBOV GP binding antibody levels to survival probability in non-human primates as an indication of vaccine-induced protection in humans [116,117]. In any case, a continued challenge is the lack of immune correlates of protection from disease caused by EBOV and filoviruses more broadly [118]. This adds complexity in establishing a strong relationship between immune response and disease outcomes [119].

Under outbreak settings, alternative regulatory mechanisms exist to expedite temporary access to vaccines. During the 2014–2016 West Africa Epidemic, WHO developed a framework coined MEURI (monitored emergency use of unregistered and experimental interventions) to allow the use of vaccines outside clinical trials [120]. As a complementary strategy, the WHO Emergency Use Listing (EUL) procedure expedites the time-limited availability of unlicensed vaccines needed in response to public health emergencies [121]. In 2017, Merck’s vaccine was recommended for deployment under an Expanded Access framework in the case of outbreaks prior to its licensure [122]. Nevertheless, obtaining a license from a stringent regulatory authority (SRA) is required to obtain WHO Pre-Qualification (PQ), which is itself a prerequisite for agencies such as UNICEF to procure vaccines on behalf of countries or to establish a global emergency stockpile. To introduce vaccines in target countries, each has the option to review dossiers through their individual National Regulatory Authorities (NRAs) or engage in a WHO-facilitated, joint assessment via mechanisms such as the African Vaccine Regulatory Forum (AVAREF) to promote harmonization with SRAs. To ensure consistent safety and efficacy, changes to the registered information of licensed products can be made through risk-based post-approval changes. These requests need to be submitted to each relevant NRA individually, leading to divergent interpretations of data and variable approval timelines [123].

### 4.7. Supply System: Manufacturing, Stockpiling, Service Delivery

A robust supply chain is crucial for the timely delivery of vaccines at the point of care. The sporadic nature of infectious disease outbreaks leads to challenges in forecasting manufacturing needs. As a result, irregular market dynamics emerge, such as unanticipated spikes in demand and short lead times to fulfill orders. Vaccines are typically manufactured across a network of facilities, each fulfilling a unique or redundant function (e.g., upstream processing of drug substance (DS), downstream processing of drug product (DP), packaging, and release). Scaling up manufacturing by adding production lines within an existing facility or scaling out to contract manufacturing organizations adds complexity to the supply chain. The model accounts for numerous parameters (e.g., processing time, batch size, failure rate, re-order point) that dictate production throughput and stockpile dynamics. Beyond production, vaccines need to be transported and distributed through multiple intermediary suppliers until the point-of-care or stored in stockpiles for future use. At each step, rigorous monitoring ensures product quality is maintained.

There is typically a gap between the total number of doses ordered and actual uptake. Understanding the heterogeneity in attitudes, sentiments, and preferences around vaccines is important to inform effective communication and better capture underlying drivers of hesitancy. As with any precautionary measure, vaccination presents a recurring tradeoff between high opportunity costs for individuals and societal benefits for populations [124]. Therefore, access to scientifically based and reliable knowledge is important for individuals to inform their behavior.

### 4.8. Costs and Benefits

While many formal approaches exist for measuring the cost-effectiveness and benefit-risk of interventions, this is outside the scope of the modeling framework presented. Nevertheless, a few stockpile-related costs (e.g., UNICEF procurement price and GAVI operational support) are included as part of the analysis to capture relative programmatic costs. The modeling approach and broad model boundaries allow greater visibility into system-wide effects that could help more accurately quantify the real value of different vaccination strategies. This supports recent efforts to broaden the boundaries of traditional cost-effectiveness analysis and metrics, including through systems analysis, to better capture complex and dynamic uncertainties in decision-making [125,126].

## 5. Results and Analysis

**Figure 5:**
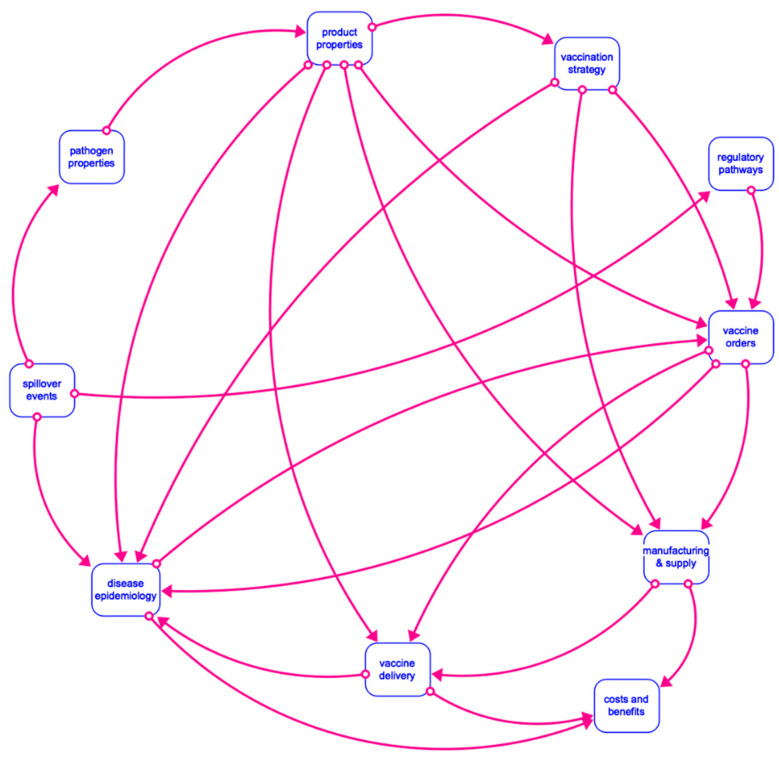
Interdependencies across model subsystems to reflect complex supply and demand dynamics driving sustainability of vaccines against EBOV.

The high-level model structure consisting of 10 interdependent subsystems is presented in Figure 5. For the purposes of this paper, most values are initially defined deterministically as a theoretical base case to show model capabilities and the impact of input variations on model KPIs. Alternatively, parameters can be defined stochastically using statistical distributions such as a truncated triangular distribution (T) defined by minimum, mostly likely, and maximum values [127]. For each setting (e.g., outbreak vs. inter-epidemic period, geographic scope), input from stakeholders is needed to further define uncertainty in key parameters. Connections between subsystems indicate where the output of one subsystem serves as an input to another (see Supplementary E). When cause-effect chains are closed, feedback loops emerge and give rise to dynamic, non-linear behavior. The results presented focus on a few critical parameters and model structures to show how the model can be used to test different leverage points. These scenarios are informed by strategic priorities and KPIs from the perspective of different stakeholders. In each case, the operational and policy relevance is described.

### 5.1. Parameter Sensitivity: Stockpile Dynamics

In the baseline scenario, the absence of spillover events means there are no vaccine orders, and proactive use is not yet considered. Therefore, stockpile dynamics are driven by the need for continuous replenishment to sustain the target level ($V_{t})$ as doses expire over time. Table 1 shows how variations in the product shelf-life and $V_{t}$, while keeping other parameters constant, have a direct impact on doses wasted and the cumulative cost to sustain the stockpile. Values reported are relative to the baseline (500,000-dose $V_{t}$ and 36 months shelf-life), with production starting at t = 630 days from the start of the simulation. In the baseline, before accounting for time-varying vaccine orders, 2 million doses are procured, and 1.5 million doses are wasted. The rates of procurement and waste are the principle drivers of financial investments required to sustain an emergency stockpile. Therefore, the model can help identify the critical price per dose to inform tendering and procurement processes. It also points to how extended product shelf-life can compensate for higher prices to avoid excess spending over the simulation period. Table 1 reports on the critical price per dose to stay within a budget of USD 150 million and 75 million, for large (500,000 doses) and small (250,000 doses) stockpiles, respectively. As Gavi’s investment strategy operates on a five-year cycle, capturing potential future costs is valuable to estimating resources that need to be allocated to sustain the stockpile.

Changing the product shelf-life does not affect the time required to initially reach the first dose and stockpile $V_{t}$, estimated to be 0.8 and 1.55 years, respectively. However, the model can evaluate the impact of alternative manufacturing processes and networks on production throughput. For example, accounting for additional losses (yield = 0.9) in the DS and DP process will lead to approximately 10% and 7.5% delay, respectively, in reaching the $V_{t}$. On the other hand, capacity expansion leads to more rapid filling of the stockpile. For example, doubling capacity could reduce the stockpile filling time by around 15%. Given the uncertainty around future outbreaks, these KPIs both contribute to enhancing system responsiveness. Minimizing the time to first dose is critical in an outbreak setting when cases grow exponentially, while reaching the $V_{t}$ contributes to preparedness in anticipation of future vaccine orders.

### 5.2. Cascading Delays: Timing of Proactive Vaccination Campaign

Multiple interdependent processes and decision points across the system can quickly accumulate into large delays. These time delays are often highly variable and depend on the epidemiologic context. Table 2 presents the impact of selected regulatory and demand-side uncertainties on the timing of initiating a proactive vaccination campaign against EBOV, assuming no constraint on manufacturing and supply. Stochastic variation in the delay associated with individual processes is represented by triangular distributions. Taking DRC as a case study, a collaborative review process is assumed following approval from an SRA and WHO pre-qualification. Additionally, the model assumes that orders can only be made at the beginning of each year. Some delays (e.g., time for NRAs to review dossiers) may not always push the start of vaccination campaigns to the following calendar year as many processes run in parallel. Decisions such as publishing updated SAGE guidance, which is a pre-requisite to activating downstream processes, are particularly influential on the campaign start time. In the baseline, in the absence of an outbreak, the first vaccination campaign is expected 1185 days from the time a dossier is submitted to the SRA. The combined effect of time-related uncertainties is a more realistic understanding of the system’s behavior as delays accumulate. Given the unpredictable nature of future outbreaks, delays leave at-risk communities vulnerable to EBOV transmission.

### 5.3. Policy Goal: Proactive Vaccine Strategies

Although current guidance around the post-license use of EBOV vaccines is focused on outbreak settings, there is growing interest in alternative strategies centered around proactive vaccination. This would involve a fundamental shift away from typical panic-and-neglect behavior to upfront investments aimed at reducing the threat of future outbreaks. However, designing a proactive vaccination program comes with many uncertainties, making it difficult to evaluate the long-term sustainability of the EBOV vaccine supply and the effectiveness of strategies. Although this paper does not seek to define an optimal intervention, the model allows users to test a broad range of trade-offs when designing a proactive program for North Kivu. Several scenarios are presented and KPIs are reported relative to an outbreak setting with 2778 cases and 1861 deaths when accounting for reactive, ring vaccination. In all scenarios, the simulation starts at $t_{o}\;$= 0 days, and the onset of the epidemic is at $t_{e}\;$ = 2000 days, while assuming a population with no prior immunity. At $t_{e}$, index cases exposed to EVD following animal-to-human transmission flow into the non-vaccinated infected stock.

Given an initial preventive vaccination order at $t_{v}$ (days), time to peak immunity following vaccination $\tau_{1}$ (days), duration of protection $\tau_{2}$ (years), onset of epidemic $t_{e}$ (days), and time between booster campaigns $t_{b}$ (years), an initial set of scenarios is defined in Table 3. Only HCWs are considered for vaccination given their elevated risk of exposure and potential to accelerate nosocomial transmission. The target coverage for HCWs is 100%. For proactive immunization, $t_{v}<t_{e}$, however, disease dynamics depends on the relative time between $t_{e}$, duration of protection $\tau_{2}$, and duration of the epidemic ($\tau_{3}$). In a one-time campaign, when $t_{b}$ is undefined, vaccination of target groups only leads to short-term gains as individuals will return to a susceptible state. From a public health perspective, $\tau_{2}$ serves as an upper-bound for $t_{b}$ to ensure sustained protection of vaccinated individuals. However, as $t_{b}$ approaches $\tau_{2}$, there is progressive waning of immunity for vaccinated individuals and a growing number of unvaccinated individuals entering the population. On the other hand, from a programmatic perspective, it may be more feasible (although potentially more costly) to integrate EVD vaccination as part of annual vaccination campaigns for HCWs.

**Figure 6:**
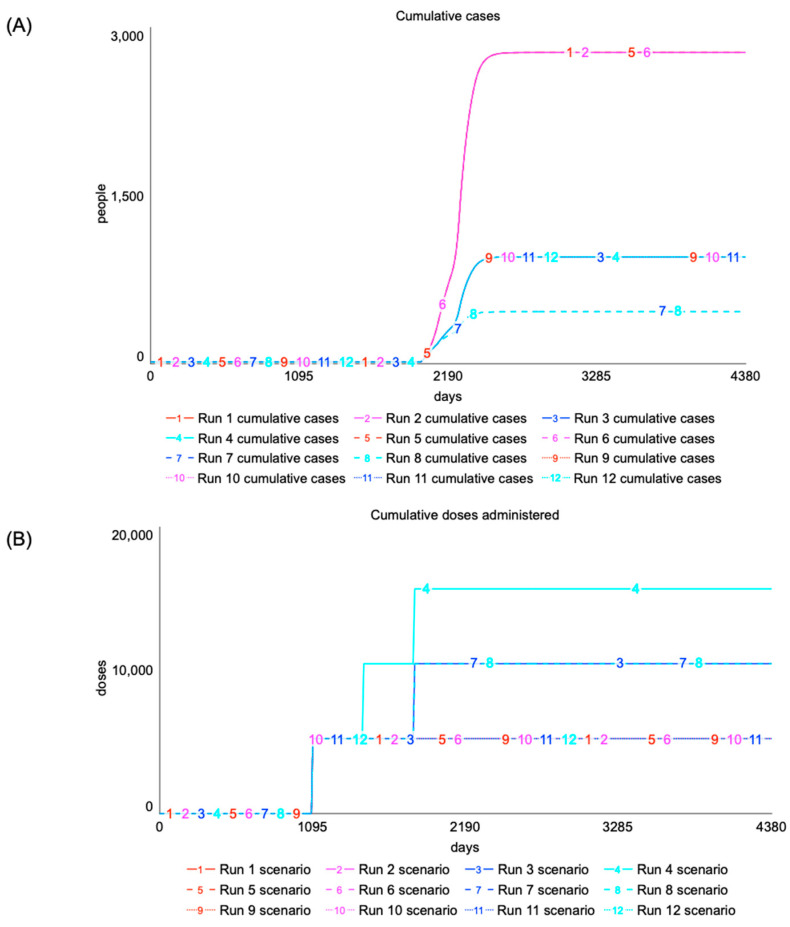
Cumulative EVD cases (**A**) and proactive EBOV doses administered (**B**) relative to reactive vaccination, accounting for uncertainty in the duration of immunity and frequency of boosters.

Results from the simulation are shown in Figure 6. Proactive vaccination has no effect when $\tau_{2}$ is small relative to the time elapsed between $t_{e}$ and $t_{v}$. Generally, disease burden is higher when the onset of an epidemic occurs prior to reaching peak immunity ($t_{v}+\tau_{1}>t_{e}$) and when it lasts longer than the protection of immunity ($t_{v}+\tau_{1}+\tau_{2}<t_{e}+\tau_{3}$). Increasing the frequency of vaccination can help close this gap, especially when $\tau_{2}$ is small. However, administering more doses does not always lead to fewer cumulative cases, as seen by scenarios 3 and 4. Protection at the time of an outbreak will depend on the relative timing between the last vaccination and $t_{e}$. Additionally, a vaccination campaign that allows sufficient time for vaccinated individuals to mount ($t_{v}+\tau_{1}<t_{e}$) and sustain ($t_{v}+\tau_{1}+\tau_{2}>t_{e}+\tau_{3}$) an immune protection is likely to have superior public health benefits. Strategies that rely on more frequent vaccination can rapidly increase the total number of vaccines that need to be ordered and administered. Although the results focus on a few scenarios in North Kivu, scaling these strategies to a broader at-risk population or other at-risk regions will put strain on the supply chain and lead to delays. On the other hand, infrequent campaigns lead to more sparsely distributed orders that could increase waste and may reduce incentives for manufacturers to continue supplying.

Taking parameter values defined in scenario 7 in Table 3, the model can be used to assess the impact of target coverage on KPIs. Although various estimates exist for the reproduction number $R_{0}$, assuming a value of 1.1 (a relatively slow-moving epidemic, as seen in North Kivu), a 70% reduction in disease severity and CFR following vaccination, and an initially naïve population, the target coverage for the general population to reach herd immunity is approximately 13%. This serves as an upper bound for the target coverage of the non-HCWs. Various scenarios are defined in Table 4 and changes in disease burden are reported relative to reactive, ring vaccination. Doses administered per case averted also serve as a proxy for the relative effectiveness of different strategies. Generally, increasing vaccine coverage for HCWs, who are at greater risk of exposure and nosocomial transmission, leads to a reasonable improvement. This is also true for the non-HCWs population, but with a decreasing effect of vaccination on disease burden as the coverage level nears herd immunity. This analysis can also be used to identify the minimum vaccine coverage to avoid saturation of the health system. Even if a strategy is less effective on a relative scale (based on the doses administered per case averted), keeping cases and hospitalizations below a critical point could avoid catastrophic economic costs and disruptions in routine care.

## 6. Discussion

Designing an effective strategy around the use of vaccines against EPPs depends on numerous dynamic and interdependent factors. This is further complicated by limited real-world evidence that makes it challenging to compare the value of different vaccination strategies. Therefore, decision-support tools need to reflect the inherent complexity of these systems to better evaluate tradeoffs. Achieving this requires overcoming disciplinary silos, meaningful stakeholder participation, and collaborative model development. Too often, the underlying drivers of delays, accumulations, and feedback loops leading to unexpected behavior are unknown or, at best, observed at a distance. To better prepare for and respond to uncertainties inherent to health emergencies, there is a need to shift from deliberated to emergent knowledge strategies [128]. In this shift, opportunities for iterative learning, model refinement, and scenario testing are abundant.

Integrating data and insights across domains and stakeholders led to the development of a novel modeling framework to assess the sustainability of vaccines against EPPs. Specifically, EBOV was used as a case study to model supply dynamics under different demand scenarios, based on user-defined vaccination strategies. Influenced by numerous factors, sustainability needs to be measured in a multi-dimensional manner. This includes the availability of vaccines and protection of at-risk populations, as well as the long-term operational and financial implications of different strategies. The subsystems presented provide a global picture of the interdependencies and uncertainties that drive timely and equitable access. Integrating perspectives, priorities, and processes from stakeholders across the system promotes discussion, a shared understanding of the problem, and the identification of high-impact leverage points for further exploration. The chosen modeling approach also facilitates a deeper, explanatory root-cause analysis of the underlying factors driving unexpected system behavior and underperformance. As a result, interventions can be co-created with stakeholders and simultaneously evaluated across multiple KPIs. Generating a broad range of scenarios allows the iterative design of interventions to meet multi-criteria objectives for sustainability. This contributes to the growing literature on vaccines against EPPs, in particular around strategic decision-making and policy design.

### 6.1. Limitations

The work presented is limited to experiences around historic EVD outbreaks. However, preparedness and response are embedded within a broader health system with continuously competing needs and priorities. The model does not reflect the extent to which vaccination strategies against EBOV could be integrated into other health services, for example, country-level Expanded Program on Immunization. This would add a valuable dimension to the model to further assess programmatic feasibility. Furthermore, each outbreak brings new challenges that can’t always be anticipated, highlighting the inherent limitations of models as simplifications of reality. As knowledge is accumulated, learnings should be reflected in model structures, especially if they are important drivers of supply sustainability. Although the model reflects perspectives across stakeholders, it does not account for competition and product prioritization. Many factors, such as the type of entity (e.g., multinational company, government, foundation) and their priorities will influence the willingness to invest in proactive EBOV vaccination strategies. Furthermore, designing a vaccination strategy or market-shaping mechanism for a proactive vaccine will inevitably impact the long-term sustainability of the reactive one. Additionally, large health emergencies can lead to competition across countries related to obtaining products first, especially in the early stages of an outbreak when supply is scarce. Beyond an individual antigen, the sustainability of vaccines against EBOV can have significant spillovers into other critical global health products in the market.

A few additional limitations are worth noting. First, demographic transitions such as aging, births, and deaths from non-EVD causes are not accounted for. Transmission dynamics are captured for different risk groups and over different periods of the epidemic. However, a limitation of compartmental models is the uniform and homogeneous mixing of individuals within each population subgroup. As a result, they fail to effectively capture more granular dynamics at the community or household level, as well as anomalies such as superspreading events. Second, non-pharmaceutical interventions (e.g., quarantines, protective equipment) and ring vaccination are not explicitly modeled as their impact on disease burden was captured in parameter calibration. However, the model could better reflect improvements in preparedness efforts (e.g., surveillance capabilities) over time, as well as the availability of post-exposure therapies. Moreover, endogenously capturing the full range of political, economic, and sociocultural factors driving behavior is beyond the scope of this work. Nevertheless, the modeling framework provides flexibility to elaborate on parameters that reflect unique contextual differences across geographies and subsets of the population. Lastly, the model focuses on disease dynamics in outbreak and inter-epidemic settings, both derived from zoonotic spillover events into human populations. However, it could also account for sexual transmission (e.g., human reservoirs), lab-based infections, and intentional biosecurity threats. This would lead to additional model structures to reflect the associated behavioral and epidemiological factors, as well as the design and testing of alternative vaccination strategies.

### 6.2. Future Research

In this paper, the emphasis is on exploring tradeoffs that emerge when considering different vaccination strategies, as well as pathogen and product-related properties. Eventually each country, in collaboration with relevant regional and global organizations, will need to design their unique policy. Therefore, continued stakeholder engagement is needed for iterative model improvement and testing of realistic scenarios. Thus far, the value of the modeling approach and its application has been validated through interviews. The development of an interactive user interface allowing users to generate and evaluate scenarios would be an important next step to validate its usability and facilitate uptake by practitioners.

Since the model provides unique insights into leverage points across the system, further modeling is needed to explore a broad range of open research questions (e.g., viral persistence in survivors, mixing two EBOV vaccines in the same geography, impact of changes in virus ecology on the risk of future spillover events). Additionally, since resources are limited and future outbreaks emerge without prior warning, priority-setting exercises can play an important role in accelerating the learning agenda.

The modeling framework could be applied to other geographic populations, risk groups, and priority pathogens. Since EBOV serves as a useful prototype pathogen, learnings could be transferred to accelerate progress on vaccine countermeasures against other viruses in the *Filoviridae* family [34,129]. This includes defining conditions necessary for the sustainability of SUDV or MARV vaccines, as well as evaluating supply and demand tradeoffs when pursuing monovalent versus broadly protective antigens.

Learnings from COVID-19 have challenged existing supply mechanisms reflected in model structures. For example, the historic concentration of R&D and manufacturing by a few entities, relying primarily on market dynamics, has led to calls for more distributed capacities driven by public health needs [130,131]. Therefore, the model could be adapted to evaluate the impact of regional manufacturing, procurement, and stockpiling strategies on the supply sustainability of vaccines against EPPs under different demand scenarios. To complement the focus on global and regional processes, growing efforts to localize preparedness and build health resilience should be explored.

The emergence of future catastrophic outbreaks due to EPPs is only a matter of time. Merely responding to acute emergencies is unlikely to be sufficient. Rather, investments in preparedness efforts that support the long-term sustainability of medical countermeasures are critical. Defining the boundaries around alternative vaccination strategies is not trivial. Therefore, decision-support tools based on available data and stakeholder experiences can help explore a broad range of scenarios to inform actions toward greater preparedness.

## Figures and Tables

**Table 1:** Sensitivity analysis of target level and product shelf-life on stockpile dynamics (grey), as well as critical procurement price per dose to stay within a given budget.

$\mathrm{Target}\;\mathrm{Level}\;V_{t}$ (Doses)	Product Shelf-Life (Months)	% Change in Doses Procured	% Change in Doses Wasted	Critical Price per Dose (USD)
500,000	36	0	0	75
	42	−18	−18	91
	48	−25	−33	100
250,000	36	−50	−50	75
	42	−55	−52	83
	48	−63	−67	100

**Table 2:** Impact of regulatory and demand-side delays on initiating a proactive vaccination campaign (grey), assuming 20 runs per scenario.

Parameter	Units	Associated Stakeholder	Baseline Value	Test Value	Average Time to Campaign (Days)	% Change Average from Baseline
Time for WHO PQ review	months	WHO	4	T(1, 4, 9)	1167	−1.5%
Time for NRA to review the dossier	months	DRC	3	T(2, 3, 12)	1185	0%
Time to publish a position paper	years	WHO	1	T(1, 2, 3)	1422	20.0%
Time to approve funding	months	GAVI	2	T(1, 2, 3)	1185	0%
Time to initiate a campaign	months	DRC	3	T(2, 3, 6)	1206	1.8%
The combined effect of variations across all parameters					1459	23.2%

**Table 3:** Impact of proactive vaccination on disease burden (grey) accounting for duration of vaccine-induced immunity and frequency of boosters.

Scenario Number	$t_{e}$ (Days)	$t_{v}$ (Days)	$\tau_{1}$ (Days)	$\tau_{2}$ (Years)	$t_{b}$ (Years)	% Change in Cases	% Change in Deaths
1	2000	1095	10	1	n/a	0	0
2	2000	1095	10	1	3	0	0
3	2000	1095	10	1	2	−65.6	−66.0
4	2000	1095	10	1	1	−65.6	−66.0
5	2000	1095	10	2	n/a	0	0
6	2000	1095	10	2	3	0	0
7	2000	1095	10	2	2	−83.3	−83.6
8	2000	1095	10	2	1	−83.3	−83.6
9	2000	1095	10	3	n/a	−65.6	−66.0
10	2000	1095	10	3	3	−65.6	−66.0
11	2000	1095	10	3	2	−65.6	−66.0
12	2000	1095	10	3	1	−65.6	−66.0

**Table 4:** Impact of proactive vaccination on disease burden (grey) accounting for target groups and coverage.

Scenario Number	Description	Target Coverage for HCWs (%)	Target Coverage for Non-HCWs (%)	% Change in Cases	% Change in Deaths	Doses Administered per Case Averted
13	Low coverage HCWs	25	0	−37.8	−37.8	2.49
14	Medium coverage HCWs	50	0	−60.8	−60.9	3.09
15	High coverage HCWs	75	0	−74.8	−75.0	3.77
16	Full coverage HCWs	100	0	−83.3	−83.7	4.52
17	Low coverage Non-HCWs	50	5	−82.2	−83.0	365.6
18	Medium coverage Non-HCWs	50	10	−84.0	−85.2	584.2
19	High coverage Non-HCWs	50	13	−84.3	−85.7	702.7
20	High coverage for both groups	100	13	−89.9	−91.4	661.2

## Data Availability

The data analysis presented in this study is available upon reasonable request from the corresponding author.

## Supplementary Materials

The following supporting information can be downloaded at: https://www.mdpi.com/article/10.3390/vaccines12010024/s1: (A) Interview guide for key-informant and in-depth interviews; (B) Interview guide for validation interviews; (C) Stakeholder interviews by organization type, geographic scope, and phase; (D) Baseline parameter values and assumptions; (E) Causal relationships between model subsystems [132,133,134,135,136,137,138,139,140,141,142,143,144,145,146,147,148,149,150,151,152,153,154].
