# Supplementary material for: Modeling Supply and Demand Dynamics of Vaccines against Epidemic-Prone Pathogens: Case Study of Ebola Virus Disease
Source: Vaccines (Basel). 2023 Dec 25;12(1):24. doi: 10.3390/vaccines12010024 (PMC10819028; doi:10.3390/vaccines12010024)
Supplement: Supplementary file 1 [file vaccines-12-00024-s001.zip › vaccines-2740248-supplementary.pdf]

Supplementary files

Supplementary A: Interview guide for key-informant and in-depth interviews

Purpose of interview:
Inform (1) our understanding of challenges across different parts of the immunization system (e.g., epidemiology, manufacturing, procurement, regulatory affairs), and (2) provide insights to help define modelling scenarios (e.g., reactive vs. proactive vaccination).

Questions:

1. UNDERSTANDING OF THE ISSUE

Ebola virus can cause deadly disease with occasional outbreaks that occur mostly in Africa. It most commonly infects animals (fruit bats, nonhuman primates) which then spillover into humans.

What are currently, from your professional perspective, the most important challenges for increasing access to vaccines against epidemic-prone pathogens in general?

- Are these challenges similar for Ebola virus?
  - How have these challenges evolved over time (for example since the West African Ebola Outbreak (2013-2016), and more recently the COVID-19 pandemic?
  - How can learnings from previous outbreaks be more systematically implemented to improve access to these types of vaccines?
- What contributes to delays in the availability of vaccines when outbreaks emerge?
  - What is an ideal response time to the first available dose (from the moment cases are reported, in anticipation of future spread and to cut transmission chains)?

2. MARKET DYNAMICS / INTERVENTIONS

Vaccines against Ebola virus have only recently been discovered and approved for use. However, given the unique market conditions – with limited demand visibility, concentration of cases in low-income regions – ensuring the available supply of safe, efficacious vaccines has been challenging.

What are ways or mechanisms to intervene in the market and de-risk investments (for industry, the national level)?

- **FINANCE / MARKET-SHAPING**:
  - What are the most important factors leading to limited willingness to invest in vaccines against epidemic-prone pathogens?
  - To what extent do subsidies (from government or foundations) for vaccine development (for developers/manufacturers) impact investment decisions?
  - Various mechanisms (e.g., advanced market commitments/advance purchase agreements, stockpiles) focus on securing demand (and supply) to reduce uncertainty and promote a steady market.
    - Why have these succeeded or failed in the past?
    - What challenges or uncertainties do you expect to see continue (or emerge)?

    - To what extent does historical return on investment of a certain product influence future, high-risk investments?

- **EPIDEMIOLOGY**: Anticipating future outbreaks to help direct preparedness efforts.
    - What factors are most likely to drive the change in the frequency/severity of future Ebola outbreaks? What is the role of improved surveillance systems?
    - The world was taken by surprise and unprepared for the 2013-2016 epidemic. Why was Ebola historically seen as having limited threat (see Jacob et al., Nature, 2020)?

- **VIROLOGY / R&D**: Considering existing Ebola vaccines licensed with WHO PQ: Ervebo (rVSV-ZEBOV) & Zabdeno/Mvabea (Ad26.ZEBOV/MVA-BN-Filo) – as well as other candidates
    - What do you see as the benefits and tradeoffs with a vaccine targeting the Zaire species of Ebola virus? Why?
    - To what extent did development of Ebola vaccines compete with other programs in the global health space?
    - How can knowledge acquired (as well as technology platforms, such as the Merck's VSV and Janssen's AdVac) help design vaccines for others in the same viral family?

- **MANUFACTURING / SUPPLY**:
    - How do the product properties (e.g., thermostability, number of doses) impact supply chain requirements? What are manufacturer and country preferences?
    - Given episodic and limited demand, what are the challenges with sustaining capabilities (e.g., technology, trained personnel) for production of Ebola vaccines?
        - Given low demand, what are losses/risks associated with stopping activities?
    - What are the prospects for regional production in Africa, where the virus is endemic?
        - What would make a technology transfer challenging?
        - Are there any adverse effects related to the prospects you have in mind?
    - What are trade-offs between having a global or regional stockpile (e.g., in Denmark compared to DRC)?

- **REGULATORY**: Understanding the regulatory process
    - What are the challenges with gaining WHO Pre-qualification (PQ)?
    - To what extent is there coordination with other national/regional entities (e.g., African Vaccine Regulatory Forum)?
    - What are challenges with gaining national registration for the vaccine (after WHO PQ) in countries at-risk of Ebola?
    - After vaccine licensure from WHO, SAGE Working Group on Ebola provides advice to WHO on how and when to use Ebola vaccines (e.g., emergency vs routine use).
        - How does WHO SAGE recommendations influence a vaccine's eligibility for GAVI funding (or other funding)?
    - How does lack of harmonization in product labeling impact production and deliveries?

- **STOCKPILING / PROCUREMENT**: Understanding the emergency stockpile established in December 2019 and facilitated by the International Coordinating Group (ICG).
    - What is the role of stockpiles in securing demand for these types of vaccines?
    - What led to a delay in the stockpile being operational (January 2021)?
    - How was the stockpile volume determined?

- What factors lead to delays in governments requesting vaccines from the stockpile?
- What are tradeoffs between stockpiling finished products vs. vaccine candidates?
- What is the value of stockpiling raw materials for vaccine production, especially if demand exceeds stockpile volumes?

3. VACCINATION STRATEGIES AND IMPLEMENTATION

Once a vaccine received regulatory authorization, it needs to be used in the most strategic and impactful way. The vaccine product profile is particularly important to inform clinical use, with implications on downstream service delivery.

How do challenges differ in terms of availability of vaccines between routine immunization and outbreaks situations and pandemics?

- How do routine vs outbreak immunization lead to different challenges?
  - What are challenges demonstrating clinical efficacy (phase 3) for episodic outbreaks?
  - How is the pace of development and testing impacted by availability of funding?
  - How could innovative vaccine trial designs or regulatory pathways help with this?
- The two WHO PQ Ebola vaccines are recommended by WHO for different use:
  - What do you see as the main tradeoffs between these two parallel strategies?
  - If it is to be used as a preventive vaccine, who are the target populations?
    - How does this compare with outbreak vaccines?
    - What is the ideal strength and duration of immunity? Why?
  - What interest is there from countries to integrate Ebola vaccine into routine immunization services/schedules? What challenges are anticipated?

4. WRAP-UP QUESTIONS

- Are there additional challenges you think should be captured (which we have not covered)?
- From all challenges we have covered in this interview, which one is currently from your experience most important to tackle first in the coming year?
- Do you have questions on the research?
  - Do you have recommendation of other people we can interview?
  - Your insights will help the design of a systems model. Would you have time for a validation session after we build the model?

Supplementary B: Interview guide for validation interviews

Purpose of interview:
Build confidence in a model, both baseline and scenarios.

Questions:

Behavior
- Does the model behave as expected?
- Does the model behavior match prior experience?
- What are deviations from expected behavior?

Structure
- What is behind this behavior?
- What is missing (or unaccounted for)?
  - Within model scope, did we miss any phenomena or views?
- Which parameters are most uncertain?
  - What is an appropriate range?
  - Most likely value? Most extreme value?
- Where do you see interventions or strategies?

Usability
- What are the most likely scenarios?
  - What other scenarios are valuable to model? Which are not relevant?
- What do you look for in a model like this?
  - Timescale, level of detail, contextual factors, etc.
- What decisions could be informed by such a model?
- How would such a model be used (e.g., within your organization)?
  - How can it be improved?
- Who could benefit from this work? Who else should we talk to?

Supplementary C: Stakeholder interviews by organization type, geographic scope, and phase

**Organization type**	**Number interviews**
Government	6
Multilateral Agency	11
Industry	15
NGOs	2
Research Organization	4
Foundation	7
Academic	1
Humanitarian Organization	3
Consulting	1
Total	***50***

**Geographic scope**	**Number interviews**
Local / National	7
Regional / Global	43
Total	***50***

**Interview phase**	**Number interviews**
Key informant & in-depth (phase 1)	
New interviewees	23
Validation (phase 2)	
Previous interviewees	12
New interviewees	15
Total	***50***

Supplementary D: Baseline parameter values and assumptions

Parameters that are dimensionless have units *dmnl.* Parameters that directly influence behavior in multiple subsystems are only documented once, in the originating subsystem.

D.1 Pathogen properties

**Parameters**	**Units**	**Values**	**Source**
Mutation switch[1]	dmnl	0	Design choice
Background rate of variation[2]	dmnl	0.03	Based on [74]
Overlap between circulating and baseline strain[3]	dmnl	n/a	n/a
Impact of viral variants on vaccine efficacy[4]	dmnl	0	Based on [74]
Viral persistence following recovery	years	2	Based on [103]
Duration of infection induced immunity	years	11	Based on [132]

[1] Defines whether viral mutations are considered in the model: 0 – off, 1 – on

[2] Stochastic parameter given uncertainty in viral evolution in zoonotic hosts prior to a spillover event and during human-to-human transmission: normal distribution with mean 0.03, standard deviation 0.025, minimum 0, and maximum 0.5

[3] In the baseline, there are no outbreaks, so it is not possible to compare circulating and baseline strains. Following a spillover event, this parameter can take non-zero values. Circulating refers to active EBOV virus in human populations, while baseline is the genetic sequence used in vaccines to encode for EBOV glycoproteins (GPs). Although not within the scope of the model, it should be noted that the two WHO pre-qualified vaccines encode for GPs from slightly different EBOV strains: Kikwit 1995 (Ervebo) and Mayinga 1976 (Zabdeno/Mvabea).

[4] Refers to the relative decrease in vaccine efficacy given the extent of variation between circulating and baseline EBOV strains. Modeled as a linear function, where the impact on efficacy increases from 0-100% as the genomic variation increases from 0-50%.

D.2 Spillover events

In the baseline model, there are no outbreaks. However, users can define the timing (day), location (country or sub-national level), and number of index cases associated with a spillover event, as well as importation of cases to neighboring regions. This subsystem serves as a bridge between the pathogen in zoonotic hosts and human populations, leading to disease spread captured in the disease epidemiology subsystem. To demonstrate use of the modeling framework in evaluating the relative impact of different leverage points on supply and demand dynamics of EBOV vaccines, only one sub-region and outbreak event is considered. This also helps with comparability of results across scenarios. Specifically, it looks at a future spillover in DRC's North Kivu province with relatively high intensity, following disease transmission similar to what was observed during the 2018-2020 epidemic in the country. In the model simulation, the number of index cases at the onset of the outbreak is assumed to be 16.

D.3 Disease epidemiology

Parameter estimation and differential equations governing transitions between disease states are based on [94], though with some important modifications. First, vaccination rates are not exogenously defined nor steady-state, but rather based on the specific vaccination strategy and time-varying availability of vaccines following orders, accounting for a broad range of potential delays. Additionally, the model adds additional flows to account for waning vaccine immunity, based on the duration of vaccine-induced immune protection. In the baseline, there are no infections, so the entire population is susceptible. At each moment in the simulation, the total population is the sum populations across all 18 compartments:

- S_{NHCW} = Susceptible population, general population other than healthcare workers (NHCW)
- S_{HCW} = Susceptible population, healthcare workers (HCW)
- V1 = Vaccinated but not yet protected HCW (prior to onset of efficacy)
- V2 = Vaccinated and protected HCW (after onset of efficacy)
- V3 = Vaccinated but not yet protected NHCW (prior to onset of efficacy)
- V4 = Vaccinated and protected NHCW (after onset of efficacy)
- E_1 = Exposed with Ebola virus, non-vaccinated or non-protected (vaccinated but prior to onset of efficacy) individuals
- I_1 = Infectious with Ebola virus, non-vaccinated or non-protected individuals
- H_1 = Infectious and hospitalized, non-vaccinated or non-protected individuals
- D_1 = Infectious and dead but not buried, non-vaccinated or non-protected individuals
- R_1 = Recovered from Ebola, non-vaccinated or non-protected individuals
- B_1 = Dead and buried, non-vaccinated or non-protected individuals
- E_2 = Exposed with Ebola virus, vaccinated individuals post onset of efficacy
- I_2 = Infectious with Ebola virus, vaccinated individuals post onset of efficacy
- H_2 = Infectious and hospitalized, vaccinated individuals post onset of efficacy
- D_2 = Infectious and dead but not buried, vaccinated individuals post onset of efficacy
- R_2 = Recovered from Ebola, vaccinated individuals post onset of efficacy
- B_2 = Dead and buried, vaccinated individuals post onset of efficacy

Parameter values for transitions in disease transmission model, adapted from [94]

**Parameter**	**Description**	**2018 DRC (North Kivu) Epidemic**					
		**Days 0–120**	**Days 120–217**	**Days 217–273**	**Days 273–385**	**Days 385–546**	**Source**
**$1/\sigma$**	Mean latency period (days)	7.0	7.0	7.0	7.0	7.0	[133]
**$1/\gamma_D$**	Mean duration from death to burial (days)	2.0	2.0	2.0	2.0	2.0	
**$1/\gamma$**	Mean duration from onset of infection to death/recovery (days)	9.6	9.6	9.6	9.6	9.6	
**$1/\alpha$**	Mean duration from onset of infection to hospitalization (days)	5.0	5.0	4.0	3.5	3.5	Fitted

**$1/\gamma_H$**	Mean duration from hospitalization to death/recovery (days)	4.6	4.6	7.0	8.0	8.2	Fitted
**$\beta_{I \to HCW}$**	Transmission rate from infectious individuals to HCW (in 1/days)	30.0	13.0	7.0	6.5	3.8	Fitted
**$\beta_{H \to HCW}$**	Transmission rate from hospitalized individuals to HCW (in 1/days)	37.0	16.0	10.0	8.0	4.2	Fitted
**$\beta_{D \to HCW}$**	Transmission rate from dead but not buried individuals to HCW (in 1/days)	0.035	0.032	0.030	0.030	0.030	Fitted
**$\beta_{I \to NHCW}$**	Transmission rate from infectious individuals to the non-HCW/general population (in 1/days)	0.2700	0.2500	0.4000	0.2780	0.2557	Fitted
**$\beta_{H \to NHCW}$**	Transmission rate from hospitalized individuals to the non-HCW/general population (in 1/days)	0.0160	0.0140	0.0200	0.0165	0.0160	Fitted
**$\beta_{D \to NHCW}$**	Transmission rate from dead but not buried individuals to the non-HCW/general population (in 1/days)	0.035	0.032	0.030	0.030	0.030	Fitted
**δ_1**	Case fatality rate among non-hospitalized infectious individuals	0.67	0.67	0.80	0.68	0.50	Fitted
**δ_2**	Case fatality rate among hospitalized individuals	0.67	0.67	0.80	0.68	0.50	Fitted

Equations defining transitions in disease transmission model, adapted from [94]

**Expressions**	**Definition**
Pulse function determined by the number of initial contacts and onset of the outbreak	Spillover from animal to human host, leading to onset of human-to-human transmission
$\left(\frac{((I_1 + \Omega I_2)\beta_{I \to HCW} + (H_1 + \Omega H_2)\beta_{H \to HCW} + (D_1 + \Omega D_2)\beta_{D \to HCW})}{N}\right) S_{HCW}$	Infection of healthcare workers by infected (non-hospitalized), hospitalized,

	and dead but not buried individuals
$\left(\frac{((I_1+\Omega I_2)\beta_{I\to NHCW}+(H_1+\Omega H_2)\beta_{H\to NHCW}+(D_1+\Omega D_2)\beta_{D\to NHCW})}{N}\right)S_{NHCW}$	Infection of the general population (non-HCW) by infected (non-hospitalized), hospitalized, and dead but not buried individuals
Pulse function determined by the number of vaccine orders and target coverage for HCWs. Rather than assuming a steady-state rate of vaccination, this defines vaccination rates based on the specific strategy and time-varying availability of vaccines following orders, accounting for a broad range of potential delays.	Vaccination of healthcare workers
Pulse function determined by the number of vaccine orders and target coverage for the general population, moving vaccine protected individuals to stock V4.	Vaccination of the general population
Delay function determined by the number of vaccinated HCWs and the time to peak vaccine induced immunity, moving vaccine protected individuals to stock V2.	Onset of efficacy of vaccine for HCWs
Delay function determined by the number of vaccinated general population and the time to peak vaccine induced immunity	Onset of efficacy of vaccine for the general population
$\frac{((I_1+\Omega I_2)\beta_{I\to HCW}+(H_1+\Omega H_2)\beta_{H\to HCW}+(D_1+\Omega D_2)\beta_{D\to HCW})V_1}{N}$ Given, Ω is the reduction in disease severity (infectiousness) for vaccine immune individuals	Infection of vaccinated healthcare workers before onset of efficacy by infected (non-hospitalized), hospitalized, and dead but not buried individuals
$\frac{((I_1+\Omega I_2)\beta_{I\to NHCW}+(H_1+\Omega H_2)\beta_{H\to NHCW}+(D_1+\Omega D_2)\beta_{D\to NHCW})V_3}{N}$	Infection of vaccinated general population (non-HCW) before onset of efficacy by infected (non-hospitalized), hospitalized, and dead but not buried individuals
σE_1	Onset of infectiousness after completion of the latency period for non-vaccinated or non-protected individuals
αI_1	Hospitalization of infectious non-vaccinated or non-protected individuals

$(1-\delta_1)\gamma I_1$	Self-recovery of infectious non-vaccinated or non-protected individuals from disease
$\delta_1\gamma I_1$	Death of infectious non-vaccinated or non-protected individuals before hospitalization
$(1-\delta_2)\gamma_H H_1$	Recovery of infectious non-vaccinated or non-protected individuals from disease after hospitalization
$\delta_2\gamma_H H_1$	Death during hospital stay for non-vaccinated or non-protected individuals
$\gamma_D D_1$	Burial/isolation of dead individuals for non-vaccinated or non-protected individuals
σE_2	Onset of infectiousness after completion of the latency period for vaccinated individuals
αI_2	Hospitalization of infectious vaccinated individuals
$(1-\mu\delta_1)\gamma I_2$<br>Given, μ is the reduction in CFR for vaccinated individuals	Self-recovery of infectious vaccinated individuals from disease
$\mu\delta_1\gamma I_2$	Death of infectious vaccinated individuals before hospitalization
$(1-\mu\delta_2)\gamma_H H_2$	Recovery of infectious vaccinated individuals from disease after hospitalization
$\mu\delta_2\gamma_H H_2$	Death during hospital stay for vaccinated individuals
$\gamma_D D_2$	Burial/isolation of dead vaccinated individuals

Conveyor stock, with a transit time equivalent to τ. People in the HCW protected stock V2 either return to S_{HCW} after τ has elapsed or are infected and go to stock E2 at a rate that is a function of the leak zone (set between 75-100%, equivalent to last quarter of the period of protection), risk of infection from EVD during outbreak, reduction in risk of infection due to vaccine protection, and size of V2 stock relative the total population.	Loss of vaccine immunity over time and infection of vaccine protected HCW
Conveyor stock, with a transit time equivalent to τ. People in the general population (non-HCW) protected stock V4 either return to S_{NHCW} after τ has elapsed or are infected and go to stock E2 at a rate that is a function of the leak zone (set between 75-100%, equivalent to last quarter of the period of protection), risk of infection from EVD during outbreak, reduction in risk of infection due to vaccine protection, and size of V4 stock relative the total population.	Loss of vaccine immunity over time and infection of vaccine protected general population (non-HCW)

Assessing model fit for disease transmission model in the absence of proactive vaccination

Source	Cumulative cases (people)	Cumulative deaths (people)
Model simulation	2778	1861
Potluri, 2022	2782	1876
WHO, 2020	2791	1875

D.4 Vaccine strategy and country orders

Determining scope of proactive vaccination campaign:

Parameters	Units	Values	Source
Total population in DRC	people	102300000	Based on [134]
Healthcare workers per 1000[5]	dmnl	1.5	Based on [135,136]
Total population in at-risk region within DRC[6]	people	8300000	Based on [137]
Target HCWs campaign coverage	dmnl	0	Design choice
Target general population campaign coverage	dmnl	0	Design choice

[5] Combination of available World Bank estimates for the number of physicians, nurses, and midwives (per 1,000 population) from 2018; scaled to levels prior to the 2018-2020 epidemic in the DRC given data used for calibration of transmission parameters.
[6] In baseline, at-risk region is defined as the North Kivu province of the DRC, where recurring outbreaks have been recorded in recent years.

Determining scope of reactive vaccination campaign:

Parameters	Units	Values	Source
Time to set up emergency stockpile	months	9	Assumption
People vaccinated per confirmed case[7]	doses	105	Based on [138,139]

Fraction of outbreak orders allocated to HCWs[7]	dmnl	0.15	Based on [138,139]
Reactive vaccines orders switch	dmnl	0	Design choice

[7] During the 2018-2020 epidemic in the DRC, approximately 3470 confirmed cases led to vaccinating close to 345,000 people using a ring vaccination protocol, while more than 21,000 others were vaccinated outside rings. Overall, approximately 15% were HCWs.

Orders for future planned proactive campaigns:

**Parameters**	**Units**	**Values**	**Source**
Expansion of healthcare workforce[8]	dmnl	0.025	Based on [140]
Attrition rate of healthcare workers[9]	dmnl	0.05	Based on [141]
Expansion of general population	dmnl	0.015	Based on [134]
Attrition rate of general population	dmnl	0.05	Assumption
Turnover time	months	6	Based on [141]
Time to publish WHO position paper	months	12	Assumption
Time for GAVI to formalize support for proactive program	months	2	Interview
Time between campaigns	years	1	Design choice
Campaign start-up time	months	3	Assumption
Number of vaccination sites	sites	6	Based on [142]
Vaccination days per week	dmnl	5	Design choice
Vaccines administered per day per site	people/site	1500	Based on [91]

[8] Assume continued effort to grow workforce, equivalent to 2.5% increase every 6 months. WHO estimates at least 2.5 medical staff (physicians, nurses and midwives) per 1,000 people are needed to provide adequate coverage of primary care interventions. This estimation would gradually increase medical staff towards the desired level by the end of the simulation period.

[9] At minimum, the incoming healthcare workers should fill the gap left from attrition.

D.5 Product properties

**Parameters**	**Units**	**Values**	**Source**
Target antigen – Ebola Zaire	dmnl	1 - yes	Based on [143,144]
Target antigen – Ebola Sudan	dmnl	0 - no	Based on [143,144]
Target antigen – Ebola Tai Forest	dmnl	0 - no	Based on [143,144]
Target antigen – Ebola Bundibugyo	dmnl	0 - no	Based on [143,144]
Target antigen – Ebola Reston	dmnl	0 - no	Based on [143,144]
Target antigen – Ebola Bombali	dmnl	0 - no	Based on [143,144]
Target antigen – Marburg	dmnl	0 - no	Based on [143,144]
Target antigen – Ravn	dmnl	0 - no	Based on [143,144]
Volume per dose	mL	1	Based on [144]
Doses per vial	doses	1	Based on [144]
Vials per pack	vials	10	Based on [144]
Number of doses for full vaccination	doses	1	Based on [144]

Minimum time between full vaccination and boost	months	6	Based on [145]
Minimum age for vaccination	years	1	Based on [144]
Maximum age for vaccination	years	n/a	Based on [144]
Dose adjustment for elderly population	dmnl	1	Based on [144]
Eligibility of pregnant women[10]	dmnl	1 - yes	Based on [143,144]
Eligibility of people living with HIV	dmnl	1 - yes	Based on [143,144]
Co-administration with other vaccines	dmnl	0 - no	Based on [143,144]
Time to peak vaccine induced immunity	days	10	Based on [113]
Duration of vaccine induced immunity[11]	years	1	Based on [146]
Risk of infection before peak vaccine induced immunity (during outbreak)	dmnl	6/1000	Based on [138]
Risk of infection after peak vaccine induced immunity (during outbreak)	dmnl	0.05/1000	Based on [138]
Reduction in case fatality ratio for vaccine immune individuals[12]	dmnl	0.7	Based on [138,147]
Reduction in disease severity for vaccine immune individuals[13]	dmnl	0.7	Assumption
Shelf life at -80°C to -60°C[14]	months	36	Based on [144]
Shelf life at 2°C to 8°C[14]	days	14	Based on [144]
Very common side effects	dmnl	9	Based on [144]
Common side effects	dmnl	7	Based on [144]
Uncommon side effects	dmnl	0	Based on [144]

[10] As a precautionary measure, it is preferable to avoid the use of EVD vaccines during pregnancy. Nevertheless, considering the severity of EVD, vaccination should not be withheld when there is a clear risk of exposure to Ebola infection.

[11] Highly uncertain given limited real-world evidence and lack of correlates of protection. Studies have shown robust antibody responses maintained up to three years post-vaccination for Ervebo [37], while persistence of primary immune response was demonstrated at least 3.8 years post-vaccination for Zabdeno/Mvabea [148].

[12] Difficult to estimate given large boundaries for case-fatality (CFR) rate across settings. For the 2018-2020 epidemic in North Kivu, the CFR was approximately 66%, with 2287 deaths from 3317 confirmed and 153 probable cases. Studies indicate improved survival following vaccination, approximated here to be 80%, leading to a CFR of 20% .

[13] Assume similar effect of vaccination on disease severity and infectiousness, as for the case fatality rate.

[14] Vaccines are initially stored and transported in an ultra-cold freezer. Once thawed, the vaccine cannot be refrozen. Upon removal from freezer, assuming more than 14 days of shelf life remain, the new expiry date comes into effect.

D.6 Regulatory pathways

Pathway for investigational products to gain a conditional license following a spillover event:

Parameters	Units	Values	Source
Time from onset of outbreak to case detection	days	30	Based on [149]
Time from case detection to declaration	days	4	Based on [149]
Time from declaration to submitting study protocols	days	14	Based on [150]
Time for WHO to receive data on candidate vaccines	days	10	Based on [150]
Time for WHO and NRA to issue import permits for investigation vaccine	days	43	Based on [150]

Pathway for investigational products to gain a full license, via WHO pre-qualification, during inter-epidemic periods:

Parameters	Units	Values	Source
Time of dossier submission to SRA	months	0	Design choice
Average time to compile dossier for submission	months	4	Interview
Accelerated review status[15]	dmnl	1	Design choice
Average SRA dossier review time	months	12	Interview
WHO PQ review time[15]	months	4	Interview
Potential countries targeted for licensure	dmnl	30	Interview
Time to set up AVAREF meetings	months	6	Interview
Fraction of countries pursuing facilitated review[16]	dmnl	0.5	Interview
Fraction of countries pursuing individual review	dmnl	0.30	Interview
Fraction of countries not pursuing review[17]	dmnl	0.20	Interview
Time to initiate facilitated review	months	2.5	Interview
Time to initiate individual review	months	2.5	Interview
Time to submit dossier to NRA (non-outbreak)[18]	days	Country-specific	Interview
Time to submit dossier to NRA (outbreak)	days	7	Interview
Time for NRA to review dossier (non-outbreak)[18]	days	Country-specific	Interview
Time for NRA to review dossier (outbreak)	days	14	Interview

[15] Alternative regulatory pathway for countermeasures with public health priority, reducing dossier review time. As an example, the US FDA's Priority Review designation reduces typical application review time to 6 months, compared to 10-12 months under standard review. Given Ervebo's dossier submission during the 2018-2020 epidemic in the DRC, an accelerate process was established for simultaneous submission to EMA, WHO, and regulatory authorities in 14 African countries, with EMA acting as the reference agency [151]. This led to WHO PQ within 36 hours of EMA approval. However, there is great variability, as the WHO PQ review took close to 9 months for Zabdeno/Mvabea.

[16] Mechanisms to support NRAs in their review process, for example, WHO's Collaborative Registration Procedure and AVAREF Joint Review Process.

[17] Approximately 20% of countries do not engage in either review path (e.g., not prioritized by product sponsor, not eligible)

[18] The regulatory subsystem is arrayed by country, so input values for each NRA need to be defined. Submission and review times are determined by many factors, including the public health context, as well

as sponsor and country priories. This leads to heterogenous timelines across countries, especially as there is currently little regional harmonization. In principle, countries engaging in facilitated review processes commit to taking a regulatory decision within an agreed timeline (e.g., 90 days), though in practice this can take much longer.

Pathway for post-approval changes:

Parameters	Units	Values	Source
Average time to review change (low risk)	months	1	Based on [152]
Average time to review change (medium risk)	months	3	Based on [152]
Average time to review change (high risk)	months	6	Based on [152]

D.7 Supply system

Manufacturing drug substance (DS), drug product (DP), and packaging/release (PR). Given the difficulty of obtaining precise data on company-specific production processes, the values presented are approximations used to generate the expected production throughput and stockpile dynamics over time.

Parameters	Units	DS	DP	PR	Source
Manufacturing switch[19]	dmnl	1	1	1	Assumption
Minimum campaign size	doses	500	500	500	Assumption
Inventory re-order point	doses	250,000	500,000	500,000	Assumption
Production startup time[20]	months	12	0	0	Interview
Time to first batch[21]	months	4	4	1.5	Assumption
Batch process time	days	14	14	3	Assumption
Baseline batch size[22]	doses	25,000	25,000	n/a	Based on [153]
Process yield[23]	dmnl	1	1	1	Assumption
Batch failure rate	dmnl	0	0	0	Assumption
Production lines	dmnl	1	1	1	Assumption
Storage temperature of product[24]	Celsius	-80	-80	-80	Interview
Minimum shelf life[25]	months	n/a	8	6	Interview
Transfer time[26]	days	n/a	2	9	Interview
Transfer loss	dmnl	0	0	0	Interview

[19] Manufacturing decision to initiate production: 0 – off, 1 – on.

[20] Time to start-up campaign before initiating first batch, including sourcing of raw materials. Assume DP and pack/release start-up time is planned or done in parallel as DS start-up. Considering a dedicated facility with continuous vaccine production, the start-up time is only accounted for ahead of the first campaign.

[21] Accounts for initial delay associated with the first batch in a campaign, including quality control and assurance steps.

[22] Accounts for typical process loss. Assume very large pack/release capacity (not a bottleneck in process), equivalent to the volume to be shipped following orders.

[23] Scaling factor for the batch size to account for additional loss or process optimization.

[24] Given limited shelf life of intermediary and finished products, this accounts for the minimum time required to initiate vaccination campaign. Doses still in inventory or stockpiles whose shelf life is below this threshold are likely to be wasted.
[25] Depending on the production network, account for transfers between facilities, for example, from DP to storage, storage to DP for packing/labelling, DP to final release facility.
[26] Fraction of doses lost due to temperature excursions during transfers between production sites.

Service delivery:

Assume doses in the pack inventory are ready to ship to country following an order. While many local factors impact the vaccine uptake, a simplified approximation is made for the vaccination rate and willingness to be vaccinated.

Parameters	Units	Values	Source
Minimum supply volume	doses	500	Assumption
Transit time from supplier to country	days	7	Interview
Storage temperature in country	Celsius	4	Assumption
Campaign setup time[27]	days	1	Assumption
Fraction of outbreak orders allocated to HCWs	dmnl	0.1	Assumption
Vaccination for HCWs switch[28]	dmnl	0	Design choice
Vaccination for general population switch	dmnl	0	Design choice

[27] Captures additional delays in setting up campaigns, though most preparations are concurrent with other processes in the system, especially during reactive vaccination.
[28] Defines whether vaccination campaigns are implemented: 0 – off, 1 – on

D.8 Costs and benefits

In the current model, only consider programmatic costs associated with sustaining a vaccine stockpile and supporting countries in implementing vaccination campaigns.

Parameters	Units	Values	Source
Procurement price per dose	USD	100	Based on [154]
Operational support cost per person targeted	USD	0.65	Interview

Supplementary E: Causal relationships between model subsystems

In the model, the value of Variable A is used as input to estimate the value of Variable B.

Variable A		→ Variable B	
**Subsystem**	**Name**	**Name**	**Subsystem**
Manufacturing & supply	Cumulative doses ordered to sustain stockpile	Cumulative cost to sustain stockpile	Cost & benefits
	Arrival rate HCWs	Arrival rate vaccines for HCWs	Vaccine delivery
	Arrival rate general	Arrival rate vaccines for general	
Vaccine delivery	Vaccines administered to HCWs	Cumulative operational support cost for campaign implementation	Costs & benefits
		Doses per case averted	
		Cumulative procurement cost for campaign implementation	
	Vaccines administered to general	Cumulative operational support cost for campaign implementation	
		Doses per case averted	
		Cumulative procurement cost for campaign implementation	
	Vaccination HCWs switch	Rate of HCWs being vaccinated	Disease epidemiology
	Vaccination general switch	Rate of general being vaccinated	
Disease epidemiology	Cumulative cases	Cost per case averted	Cost & benefits
	Cumulative HCWs vaccinated	Doses administered per case averted	Cost & benefit
	Cumulative general vaccinated		
	Baseline number of cases	Doses per case averted	
	Rate of EBOV infections (cases)	Rate of ordering for reactive campaigns	Vaccine orders
Vaccine orders	Order for HCWs for proactive campaigns	Total doses requested by DRC for HCWs	Manufacturing & supply
	Orders for general for proactive campaigns	Total doses requested by DRC for general	
	Time to set up emergency stockpile	Drug substance (DS) order rate	
	Campaign start up time	WIP HCW campaign	Vaccine delivery
	Orders for HCWs for proactive campaigns	Rate of HCWs being vaccinated	Disease epidemiology
	Orders for general for proactive campaigns	Rate of general being vaccinated	
	Time between campaigns	Vaccine immune HCWs	

		Vaccine immune general	
	Campaign start up time	Vaccine immune HCWs	
Spillover events	Index cases outbreak	Rate of non-vaccinated being infected through a spillover event	
	Time onset of outbreak	Rate of non-vaccinated being infected through a spillover event	
	Index cases outbreak	Overlap between circulating and baseline strain	Pathogen properties
	Country of outbreak	Emergency submission in IR & JR country	Regulatory pathways
		Emergency review in IR & JR country	
Regulatory pathways	Country license from joint review	Rate of putting in proactive orders for HCWs	Vaccine orders
	Realized time to SRA approval	WHO SAGE guidance	
		Funds available	
	Time of dossier submission to SRA	WHO SAGE guidance	
		Funds available	
	WHO PQ review time	WHO SAGE guidance	
		Funds available	
Pathogen properties	Impact of viral variants on vaccine efficacy	Actual reduction in risk of infection due to vaccine protection	Product properties
		Actual reduction in disease severity for vaccine immune individuals	
Product properties	Baseline reduction in disease severity for vaccine immune individuals	Rate of exposure of vaccinated (not yet immune) HCWs	Disease epidemiology
		Rate of exposure of susceptible HCWs	
		Rate of exposure of vaccinated (not yet immune) general	
		Rate of exposure of susceptible general	
	Duration of vaccine induced immunity	Vaccine immune HCWs	
		Vaccine immune general	
	Reduction in case fatality ratio for vaccine immune individuals	Rate of vaccine immune hospitalized dying	
		Rate of vaccine immune infected dying, without hospitalization	

		Rate of vaccine immune infected recovering	
		Rate of vaccine immune hospitalized recovering	
	Risk of infection before peak vaccine induced immunity	Rate of vaccine immune general being exposed	
		Rate of vaccine immune HCWs being exposed	
	Time to peak vaccine induced immunity	Rate of vaccinated general becoming immune	
		Rate of vaccinated HCWs becoming immune	
	Shelf life at -80C	Drug Product (DP) waste	Manufacturing and supply
	Duration of vaccine induced immunity	Rate of immune healthcare workers becoming non-immune	Vaccine orders
		Rate of immune general becoming non-immune	
	Number of doses for full vaccination	Order for HCWs for proactive campaigns	
	Time between prime and boost dose for two dose vaccine	Rate of general in queue being vaccinated	
		Rate of healthcare workers in queue being vaccinated	
	Time to peak vaccine induced immunity	Rate of general in queue being vaccinated	
		Rate of healthcare workers in queue being vaccinated	
	Minimum age for vaccination	Actual general campaign coverage	Vaccine strategy
	Zaire	Actual HCW campaign coverage	
		Actual general campaign coverage	
	Impact of perceived safety risk on uptake	Vaccine uptake for general	Vaccine delivery
	Shelf life at -80C	Shelf life in country	
	Shelf life at 4C		
Vaccine strategy	HCWs in at risk provinces	Susceptible HCWs	Disease epidemiology
	General population in at risk provinces	Susceptible general	
	Target HCWs in at risk provinces	Susceptible HCWs being vaccinated	

	Target general in at risk provinces	Susceptible general being vaccinated	
	Doses per confirmed case	Orders for reactive campaigns	Vaccine orders
	Target HCWs in at risk provinces	Non-immune healthcare workers	
	Target general in at risk provinces	Non-immune general	
